# ^18^F-FDG PET Combined With MR Spectroscopy Elucidates the Progressive Metabolic Cerebral Alterations After Blast-Induced Mild Traumatic Brain Injury in Rats

**DOI:** 10.3389/fnins.2021.593723

**Published:** 2021-03-18

**Authors:** Yang Li, Kaijun Liu, Chang Li, Yu Guo, Jingqin Fang, Haipeng Tong, Yi Tang, Junfeng Zhang, Jinju Sun, Fangyang Jiao, Qianhui Zhang, Rongbing Jin, Kunlin Xiong, Xiao Chen

**Affiliations:** ^1^Department of Nuclear Medicine, Daping Hospital, Army Medical University, Chongqing, China; ^2^Department of Radiology, Daping Hospital, Army Medical University, Chongqing, China; ^3^Department of Medical Imaging, Air Force Hospital of Western Theater Command, Chengdu, China; ^4^Department of Gastroenterology, Daping Hospital, Army Medical University, Chongqing, China; ^5^Department of Foreign Language, Army Medical University, Chongqing, China; ^6^Chongqing Clinical Research Center for Imaging and Nuclear Medicine, Chongqing, China

**Keywords:** blast injury, mild traumatic brain injury, positron emission tomography, fluorodeoxyglucose, magnetic resonance spectroscopy

## Abstract

A majority of blast-induced mild traumatic brain injury (mTBI) patients experience persistent neurological dysfunction with no findings on conventional structural MR imaging. It is urgent to develop advanced imaging modalities to detect and understand the pathophysiology of blast-induced mTBI. Fluorine-18 fluorodeoxyglucose positron emission tomography (^18^F-FDG PET) could detect neuronal function and activity of the injured brain, while MR spectroscopy provides complementary information and assesses metabolic irregularities following injury. This study aims to investigate the effectiveness of combining ^18^F-FDG PET with MR spectroscopy to evaluate acute and subacute metabolic cerebral alterations caused by blast-induced mTBI. Thirty-two adult male Sprague–Dawley rats were exposed to a single blast (mTBI group) and 32 rats were not exposed to the blast (sham group), followed by ^18^F-FDG PET, MRI, and histological evaluation at baseline, 1–3 h, 1 day, and 7 days post-injury in three separate cohorts. ^18^F-FDG uptake showed a transient increase in the amygdala and somatosensory cortex, followed by a gradual return to baseline from day 1 to 7 days post-injury and a continuous rise in the motor cortex. In contrast, decreased ^18^F-FDG uptake was seen in the midbrain structures (inferior and superior colliculus). Analysis of MR spectroscopy showed that inflammation marker myo-inositol (Ins), oxidative stress marker glutamine + glutamate (Glx), and hypoxia marker lactate (Lac) levels markedly elevated over time in the somatosensory cortex, while the major osmolyte taurine (Tau) level immediately increased at 1–3 h and 1 day, and then returned to sham level on 7 days post-injury, which could be due to the disruption of the blood–brain barrier. Increased ^18^F-FDG uptake and elevated Ins and Glx levels over time were confirmed by histology analysis which showed increased microglial activation and gliosis in the frontal cortex. These results suggest that ^18^F-FDG PET and MR spectroscopy can be used together to reflect more comprehensive neuropathological alterations *in vivo*, which could improve our understanding of the complex alterations in the brain after blast-induced mTBI.

## Introduction

Blast-induced traumatic brain injury (TBI), the most common injury of modern warfare, has been receiving great interest worldwide recently (Benzinger et al., 2009). The nature of modern warfare and the frequent use of improvised explosive devices (IEDs) have led to increases in mild TBI (mTBI), which is defined as loss of consciousness lasting less than 30 min, an initial Glasgow Coma Score (GCS) of 13–15, and posttraumatic amnesia lasting less than 24 h (Bailes and Cantu, 2001; Belanger et al., 2007). Even though the acute symptoms of mTBI may be mild and transient, experimental and clinical studies have found metabolic, biochemical, and structural changes caused by mTBI. Mild TBI causes a complex pathophysiological cascade, including dramatic alterations in ionic homeostasis (Katayama et al., 1991), disruption of the blood–brain barrier (BBB) (Walls et al., 2016; Kuriakose et al., 2018), injury-induced neuroinflammation (Kokiko-Cochran and Godbout, 2018; Missault et al., 2019), and diffuse axonal injury (Ling et al., 2012; Venkatasubramanian et al., 2020). However, majority of mTBI patients experience neurological dysfunction with no findings on conventional clinical imaging methods, such as structural magnetic resonance imaging (MRI) or computed tomography (CT) (Le and Gean, 2009). Due to the difficulty of imaging assessment on mTBI patients, especially in the acute or subacute phase, there needs more emphasis on the development of advanced imaging modalities, so that the therapeutic management of mTBI patients can be improved.

Several advanced *in vivo* imaging techniques have been used to investigate TBI to better understand the temporal microstructural and functional changes following TBI, such as diffusion tensor imaging (DTI), functional MRI (fMRI), MR spectroscopy (MRS), positron emission tomography (PET), etc. (Croall et al., 2014; Zhuo et al., 2015; Harris et al., 2016; Jaiswal et al., 2019; Kulkarni et al., 2019; Venkatasubramanian et al., 2020). DTI has been widely used to investigate the microstructural responses after mTBI. Many different regions have been found to be affected, since DTI is highly sensitive to axonal damage related to mTBI (Mayer et al., 2010; Tang et al., 2017; Badea et al., 2018; Venkatasubramanian et al., 2020). In contrast to the structural information offered by DTI regarding brain integrity, some studies have explored functional and biochemical changes following brain injury using other imaging modalities. Fluorine-18 fluorodeoxyglucose PET (^18^F-FDG PET) is a powerful imaging technique that can map regional cerebral metabolism patterns *in vivo*. Cerebral glucose metabolism significantly associates with neuronal function and activity of the injured brain (Garnett et al., 2001; Casey et al., 2008). Clinical and animal studies have shown that changes in glucose metabolism of the brain in hours to days following exposure to blast injury have been more complex than the chronic hypometabolism using ^18^F-FDG PET (Stocker et al., 2014; Awwad et al., 2015; Meabon et al., 2016; Jaiswal et al., 2019). PET imaging provides exquisite sensitivity when compared with CT or MR imaging, but with reduced resolution. *In vivo* MRS provides complementary information and assesses metabolic irregularities following injury. Metabolites such as *N*-acetylaspartate (NAA), choline (Cho), and lactate (Lac), glutamate and glutamine (Glx), myo-inositol (Ins), and taurine (Tau) detected by ^1^H MRS provide information related to brain injury, inflammation, ischemia, and mitochondrial dysfunction (Xu et al., 2011; Harris J.L. et al., 2012; Sajja et al., 2012; Zhuo et al., 2015). ^18^F-FDG PET and MRS could be used to investigate the neuropathological changes after brain injury from different aspects. However, it is currently unclear if ^18^F-FDG PET could be used synergistically with MRS to elucidate the acute pathological alterations in the brain and improve diagnostic and prognostic measures after blast-induced mTBI.

In this study, we aim to evaluate hyperacute, acute, and subacute metabolic cerebral alterations caused by blast-induced mTBI in rats *via* the combination of *in vivo* MRS and ^18^F-FDG PET. Animals were exposed to a single blast overpressure wave, underwent MRS and ^18^F-FDG PET, and sacrificed for histological analysis at baseline, 1–3 h post-injury (hyperacute phase), 1 day post-injury (acute phase), and 7 days post-injury (subacute phase). Combining these two imaging methods could reflect more comprehensive neuropathological alterations *in vivo* after blast-induced mTBI.

## Materials and Methods

### Animals

Sixty-four adult male Sprague–Dawley rats (obtained from the Experimental Animal Center of Daping Hospital, Chongqing, China) weighing 200∼225 g were randomly assigned into two groups, namely the mTBI group (*n* = 32) and the sham group (*n* = 32). Throughout the experiment, rats were kept in a temperature- and humidity-controlled room under a 12-h light/12-h dark cycle with food and water except during ^18^F-FDG uptake. All animal procedures used in this study were approved by the Administration of Affairs Concerning Experimental Animals Guideline of Army Medical University. The use of laboratory animals was in compliance with the guidelines of National Institutes of Health. All animal experiments were approved by the Animal Use Subcommittee of Army Medical University.

### Experimental Design

**FIGURE 1:**
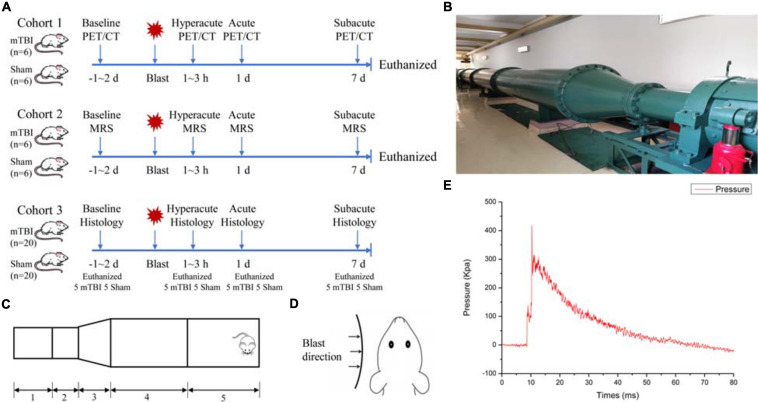
Schematic drawing of the experimental protocol and bio-shock tubes (BST-I). **(A)** Overall experimental scheme for blast-induced mild traumatic brain injury (mTBI) and sham rats. **(B)** The diagram of the BST-I shock tube. **(C)** BST-I model: (1) driving section, (2) double-clamping diaphragm section, (3) conical section, (4) transition section, and (5) test section. **(D)** Blast was directed to the left side of all rats. **(E)** Representative blast waves from a single blast illustrating peak pressure values of 139.146 and 364.86 kPa, lasting 48.41 ms.

Figure 1A shows the experimental scheme for animals in this study. The study contained three cohorts of adult male Sprague–Dawley rats. The first cohort received blast-induced mTBI (*n* = 6) or sham (*n* = 6) and underwent PET/CT scans with ^18^F-FDG prior to injury and at 1–3 h, 1 day, and 7 days post-injury. The second cohort received blast-induced mTBI (*n* = 6) or sham (*n* = 6) and underwent MRI scans including conventional structure imaging and MRS prior to injury and at 1–3 h, 1 day, and 7 days post-injury. The last cohort received blast-induced mTBI (*n* = 20) or sham (*n* = 20) and were sacrificed for Evans blue penetration assay and immunohistochemical analysis prior to injury and at 1–3 h, 1 day, and 7 days post-injury.

### Blast-Induced mTBI Model

The bio-shock tube (BST-I) apparatus (Figures 1B,C) was used to produce blast injury as previously described (Ning et al., 2019). All rats were anesthetized in an induction chamber with 4–5% isoflurane in 100% oxygen for approximately 5 min. Then, the anesthetized rats in the mTBI group were placed into individual cages (one rat/cage) in the prone position with the left side facing the direction of the membrane during blast (Figure 1D). All cages were positioned at the same vertical plane to ensure equal pressure exposure and to prevent subsequent secondary or tertiary blast injuries. The BST-I apparatus generated two levels of lower-intensity blast waves. The values for the peak overpressure were 139.146 and 364.86 kPa, lasting 48.41 ms (Figure 1E). Following the blast, the rats were removed from the apparatus and placed in a supine position in a cage. Sham rats went through the same procedure of anesthesia and were placed in the BST-I apparatus without being exposed to the blast waves. The righting reflex time was recorded for both mTBI and sham rats.

### PET/CT Imaging, Reconstruction, and Analysis

Rats were food-deprived 4∼6 h before ^18^F-FDG injection. ^18^F-FDG was injected intravenously *via* the tail vein to both mTBI and sham rats with the dose of 37 MBq/rat. After 40 min of uptake, static PET scan was performed for 30 min in list mode (350∼650 keV, 2.56 ns) using a small animal micro PET/CT scanner (Pingseng, Jiangsu, China). A three-bed CT scan was acquired in rat mode (60 kVp, 500 μA, exposure time 320 ms) following PET scans for anatomical localization, attenuation, and scatter corrections. The CT image matrix was 352 × 352 × 536 with a voxel size of 0.23 × 0.23 × 0.23 mm. The parameters for reconstruction were as follows: reconstruction algorithm = 3D-OSEM/MAP with 2 OSEM iterations and 18 MAP iterations; scatter, attenuation, and decay corrections applied; requested resolution = 0.5 mm; image matrix = 256 × 256 × 159; and voxel size = 0.39 × 0.39 × 0.80 mm. The intrinsic resolution of the PET scanner was approximately 1.4 mm full width at half maximum (FWHM) at the center of the field of view.

Processing and analysis of ^18^F-FDG PET data was performed using PMOD software version 3.6 (PMOD Technologies, Ltd., Zurich, Switzerland) as previously described (Park et al., 2019). For volume of interest (VOI) analysis, the PET data were registered to the 58 regions in the W. Schiffer rat brain template and atlas by PMOD (Supplementary Table 1). The standard uptake value (SUV) in defined subregions of the rat brain was automatically applied to measure. This study evaluated 58 brain regions and the entire atlas was used for whole brain normalization as previously reported (Byrnes et al., 2014). The regional SUVw was calculated by dividing the standardized ^18^F-FDG uptake value for the individual target region by that for the whole brain.

### MR Imaging, Processing, and Analysis

MR imaging experiments were conducted before and at 1–3 h, 1 day, and 7 days after blast in the mTBI and sham groups. All MR imaging experiments were performed with 7 T animal scanner (Biospin70/20 USR, Bruker BioSpin, Ettlingen, Germany) equipped with a four-channel rat head transmitter/receiver coil. Rats were initially anesthetized in a box with 5% isoflurane in oxygen, then anesthesia was maintained by 2% isoflurane in oxygen during MR scanning. The respiration and heart rate were monitored throughout the experiment. Scanning sequences included T1-weighted imaging (Turbo-RARE, echo time/repetition time: 6.5 ms/800 ms, field of view: 30 × 30 mm, matrix: 256 × 256, slice thickness: 1 mm), T2-weighted imaging (Turbo-RARE, echo time/repetition time: 30 ms/3,000 ms, field of view: 30 × 30 mm, matrix: 256 × 256, slice thickness: 1 mm), and MRS (STEAM, echo time/repetition time: 3 ms/3.5 ms, a single 2 × 2 × 2 mm^3^ voxel was located in the left somatosensory cortex region based on T2-weighted images). The MRS voxels were localized in the somatosensory cortex and hippocampus region of the rat brain. These regions were chosen because they were implicated in previous studies using other TBI models (Larson et al., 2011; Sajja et al., 2012; Awwad et al., 2015; Brabazon et al., 2017; Salberg et al., 2017; Tang et al., 2017). MRS data were analyzed by jMRUI software 5.2, and the method of parameterization implemented in jMRUI software was applied: advanced method for accurate, robust, and efficient spectral fitting (AMARES). The AMARES algorithm estimates the area under each peak based on the frequency and half-width of each peak (Vanhamme et al., 1997), and the relative levels of Ins, Tau, Cho, Glx, Glu, NAA, and Lac to creatine (Cr) were assessed. The Cr spectral intensity was used as reference as previously reported for relative quantitation because of its relatively stable concentration in the brain (Xu et al., 2011).

### Evans Blue Penetration Assay

The permeability of the BBB was determined by measuring the penetration of Evans blue (Solarbio, Beijing, China) in the brain tissues of three rats in each group at each time point. Evans blue (2% in saline; 4 ml/kg body weight) was injected intravenously *via* the tail vein 1 h before measurement. The anesthetized rats were perfused transcardially with saline before sampling. Photos of the brains were taken with a digital camera. Each sample was weighed and homogenized with 400 μl PBS, then precipitated by 60% trichloroacetic acid overnight. The sample was centrifuged for 30 min at 10,000 rpm. Absorption of the supernatant was measured at a wavelength of 620 nm with a plate reader. The extravasation of Evans blue was quantified as microgram/gram brain tissue with an Evans blue standardized curve.

### Immunohistochemical Evaluation

For histological analysis of brain tissue, two rats in each group at each time point were euthanized, then perfused with 4% paraformaldehyde followed by saline. The entire brain was collected and fixed in 4% paraformaldehyde. After being fixed for over 48 h, the brain tissues were embedded in paraffin. Brain tissue was sectioned at 20 μm. Hematoxylin/eosin (H&E) staining on paraffin sections was histologically reviewed. For immunochemistry, Vectastain Elite ABC Kit (Vector Laboratories, Burlingame, CA, United States) and DAB Peroxidase Substrate Kit (Vector Laboratories, Burlingame, CA, United States) were used with primary antibodies, namely anti-rat Iba1 (ab178846, Abcam, Cambridge, MA, United States), anti-rat GFAP (ab7260, Abcam, Cambridge, MA, United States), and anti-rat NeuN (ab177487, Abcam, Cambridge, MA, United States), followed by biotinylated goat anti-rabbit and anti-mouse IgG that were used as secondary antibodies (Vector Laboratories, Burlingame, CA, United States). Images were then collected with an Olympus BX43 microscope (Olympus America, Center Valley, PA, United States). We examined entire sections at × 40 magnification to identify “hot spot” regions of protein expression within the frontal cortex and hippocampus, from which five hot spots were selected for imaging. Immunoreactivity of these proteins was evaluated using the mean optical density (IOD/area) of protein expressions at × 200 magnification field by Image-Pro Plus 6.0 (Media Cybernetics, Rockville, MD, United States).

### Statistical Analysis

All data are presented as mean ± standard deviation (SD). For the PET, MRS, and immunohistochemical data, two-way analysis of variance (ANOVA) with repeated measures was used to analyze alterations in ^18^F-FDG uptake, various neurometabolites, Evans blue, and immunohistochemical index (IOD/area values) over time. Individual comparisons at each time point or different groups were conducted with Sidak’s multiple comparisons test. All statistical analyses were performed using GraphPad Prism 8 (GraphPad Software, San Diego, CA, United States). Two-tailed *p* values were calculated with the significance level at 0.05.

## Results

### Trauma Induction

All rats survived the blast, without observable skull fracture and apnea. Both sham and mTBI groups had similar righting reflex time, with the average time of 296.2 ± 35.4 s for the sham group and 325.7 ± 40.2 s for the mTBI group. There were no significant differences in size and weight between these two groups at any time points after the blast. Moreover, rats in neither group showed any visible sign of injury in the brain on T1- and T2-weighted images at any time points after the blast (Supplementary Figure 1).

### ^18^F-FDG PET Detected Both Increased and Decreased Brain Metabolism in Multiple Regions After Blast-Induced mTBI

**FIGURE 2:**
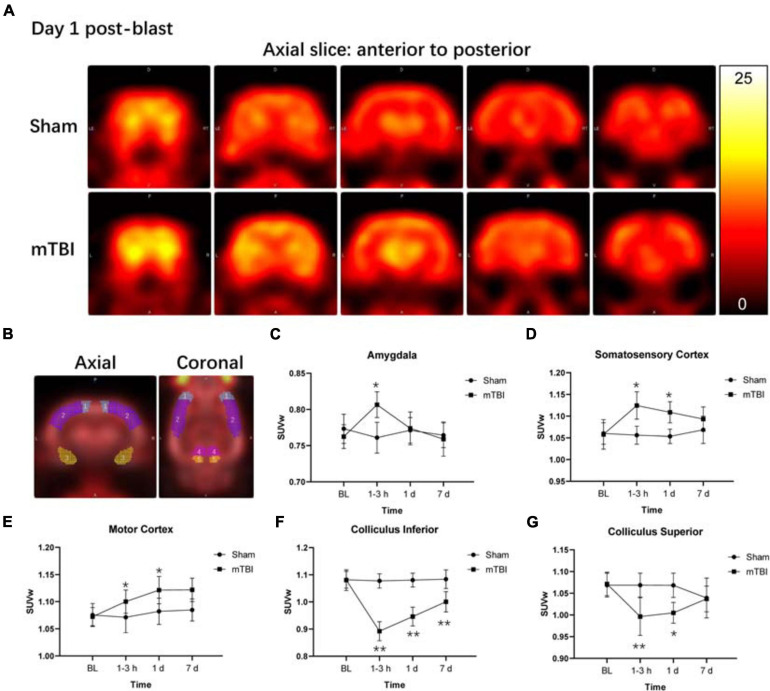
Fluorine-18 fluorodeoxyglucose positron emission tomography (^18^F-FDG PET) reveals both increased and decreased brain metabolism in multiple regions after blast-induced mTBI. **(A)** Representative axial ^18^F-FDG PET images in the brain from anterior to posterior (left to right) of a sham (up) and a blast (bottom) rat on day 1 post-blast. In the corresponding PET images, yellow and black represent relative higher and lower values, respectively. **(B)** Volume-based analysis of ^18^F-FDG uptake showing five regions with significant changes between sham and mTBI rats: (1) motor cortex, (2) somatosensory cortex, (3) amygdala, (4) colliculus superior, and (5) colliculus inferior. **(C–G)** Box plots show ^18^F-FDG uptake at 1–3 h, 1 day, and 7 days post-injury in these five regions of mTBI and sham rats. Box and whisker plots display median, first and third quartiles, minimum, and maximum. **p < 0.01 vs. the sham group; *p < 0.05 vs. the sham group.

Among 58 brain regions, 10 VOIs were found significantly changed between sham and mTBI rats in ^18^F-FDG uptake from baseline to day 7, including the bilateral amygdala (*F*_1,5_ = 4.137, *p* = 0.0253), somatosensory cortex (*F*_1,5_ = 13.43, *p* = 0.0145), motor cortex (*F*_1,5_ = 7.479, *p* = 0.0410), colliculus inferior (*F*_1,5_ = 38.77, *p* = 0.0016), and colliculus superior (*F*_1,5_ = 16.82, *p* = 0.0093) (Figure 2B and Supplementary Table 2). As shown in Supplementary Table 2, the changes of ^18^F-FDG uptake in bilateral VOIs were similar, so that we combined SUVw of the left and right VOIs as SUVw average to be compared at different time points. At 1–3 h post-injury, the amygdala (+ 5.97%, *p* = 0.0103) and somatosensory cortex (+ 6.82%, *p* = 0.0245) presented increased ^18^F-FDG uptake in the mTBI group, compared with the sham group. Subsequently, ^18^F-FDG uptake in the amygdala and somatosensory cortex gradually returned to baseline from 1 to 7 days post-injury in the mTBI group, with a significant difference in somatosensory cortex by 1 day (+ 6.12%, *p* = 0.0429) compared with the sham group (Figures 2A,C,D). ^18^F-FDG uptake in the motor cortex increased in the mTBI group from 1 day (+ 3.60%, *p* = 0.0326) to 7 days post-injury (+ 3.41%, *p* = 0.0446) (Figures 2A,E). However, at 1–3 h post-injury, decreased ^18^F-FDG uptake was observed in two midbrain structures, i.e., the inferior colliculus (-17.21%, *p* < 0.0001) and superior colliculus (-6.75%, *p* = 0.0094). Then, ^18^F-FDG uptake in the superior colliculus and inferior colliculus gradually returned to baseline from 1 to 7 days post-injury in the mTBI group, with a significant difference in the inferior colliculus by 1 day (-12.43%, *p* < 0.0001) and 7 days (-7.68%, *p* = 0.0049) and in the superior colliculus by 1 day (-5.95%, *p* = 0.0230) compared with the sham group (Figures 2A,F,G).

### MRS Revealed *in vivo* Dynamic Neurochemical Alterations in the Cortex Region After Blast-Induced mTBI

**FIGURE 3:**
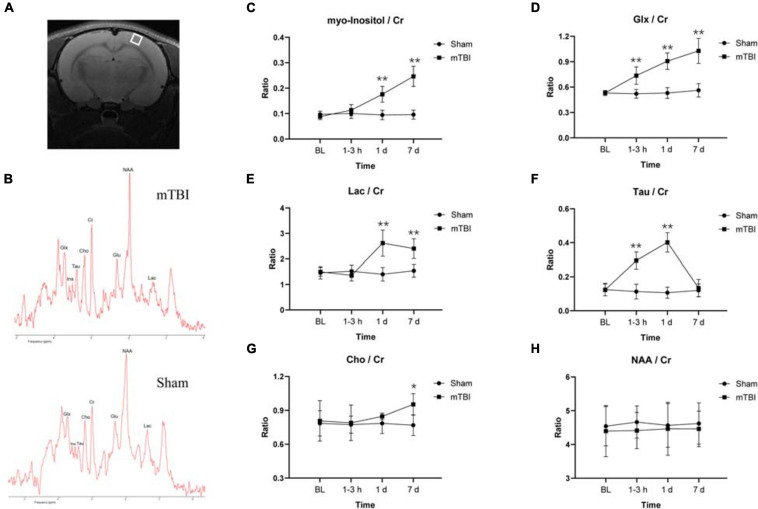
Magnetic resonance spectroscopy (MRS) detects *in vivo* dynamic neurochemical alterations in the somatosensory cortex region after blast-induced mTBI. Placement of voxel in the somatosensory cortex region [white box **(A)**] with a representative spectrum (up: mTBI 1–3 h post-injury; bottom: sham) **(B)**. **(C–H)** Box plots show the relative levels of Ins, Glx, Lac, Tau, Cho, and NAA at 1–3 h, 1 day, and 7 days post-injury in somatosensory cortex of mTBI and sham rats. Box and whisker plots display median, first and third quartiles, minimum, and maximum. **p < 0.01 vs. the sham group; *p < 0.05 vs. the sham group. Ins, myo-inositol; Tau, taurine; Cho, choline; Glx, glutamine + glutamate; Glu, glutamate; NAA, *N*-acetylaspartate; Lac, lactate; Cr, creatine.

The MRS voxel was placed to the somatosensory cortex (Figure 3A) and hippocampus (Supplementary Figure 2A) of rat brain. High-quality spectra with narrow linewidths were generally obtained in this study (Figure 3B and Supplementary Figure 2B). For the somatosensory cortex, MRS revealed relative levels of glia marker Ins (*F*_1,5_ = 54.49, *p* = 0.0007), oxidative stress and gliosis marker Glx (*F*_1,5_ = 31.38, *p* = 0.0025), and hypoxic indicator Lac markedly elevated as early as 1–3 h and day 1 post-injury, together with day 7 (Figures 3C–E). The major osmolyte, the relative level of Tau (*F*_1,5_ = 21.90, *p* = 0.0054), immediately increased as early as 1–3 h (+ 160.37%, *p* < 0.0001), continuously elevated by 1 day (+ 276.48%, *p* < 0.0001), but decreased to sham level on 7 days post-injury (+ 11.92%, *p* = 0.9444) (Figure 3F). The mTBI group showed a significant increased relative level of Cho on 7 days post-injury, compared with the sham group (+ 24.04%, *p* = 0.0366) (Figure 3G). No significant reduction in the relative level of NAA was found in the mTBI group, compared with the sham group at each time point (*F*_1,5_ = 0.9721, *p* = 0.3694) (Figure 3H). However, no significant differences of these metabolites, namely Ins (*F*_1,5_ = 3.218, *p* = 0.1328), Glx (*F*_1,5_ = 2.068, *p* = 0.2099), Lac *(F*_1,5_ = 1.860, *p* = 0.2308), Tau (*F*_1,5_ = 0.03899, *p* = 0.8512), Cho (*F*_1,5_ = 1.628, *p* = 0.2580), and NAA (*F*_1,5_ = 0.7159, *p* = 0.4361), were observed in the hippocampus between the mTBI group and the sham group (Supplementary Figures 2C–H).

### Blast-Induced mTBI and Immunohistochemical Markers of Inflammation, Injury, and Astrogliosis

**FIGURE 4:**
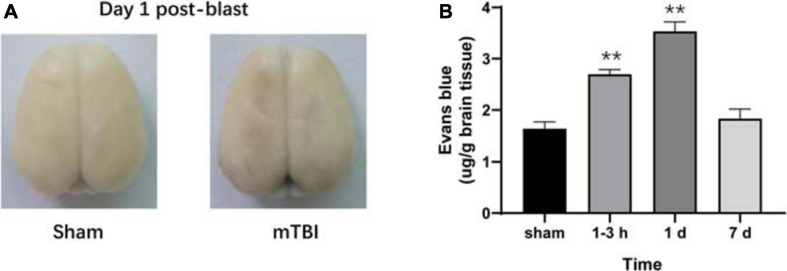
Evans blue (EB) penetration assay reveals progressive alterations of the blood–brain barrier (BBB) in rats after blast-induced mTBI. **(A)** Representative EB dyeing images of a sham (left) and a blast (right) rat on day 1 post-blast. **(B)** EB content in brain tissue was also quantified and expressed as micrograms of EB per gram of brain tissue. **p < 0.001 vs. the sham group.

**FIGURE 5:**
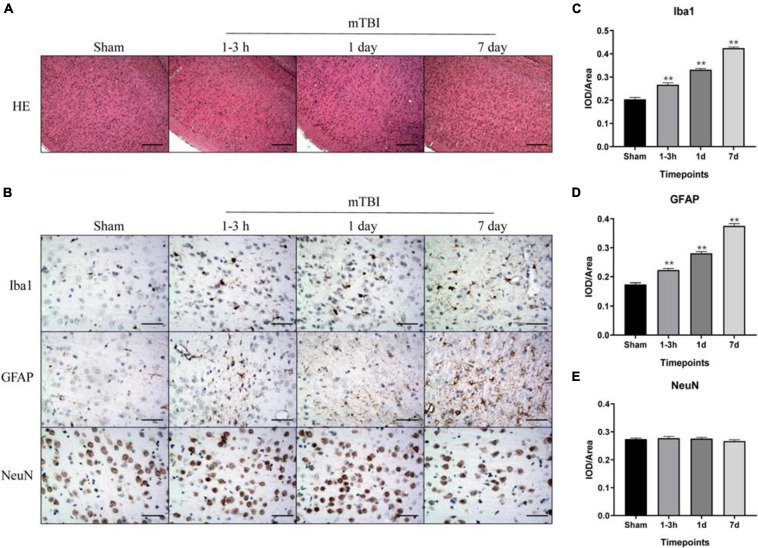
Immunohistochemical analysis of inflammation, injury, and astrogliosis in the frontal cortex region after blast-induced mTBI. **(A)** H&E staining shows no obvious lesion after exposure to blast at each time point in mTBI rats. **(B)** Representative immunohistochemical staining for Iba 1, GFAP, and NeuN expression in the frontal cortex region is shown at each time point following injury in sham and mTBI rats. **(C–E)** Iba 1, GFAP, and NeuN expression levels were quantified. The scale bar denotes 250 μm in **(A)** and 50 μm in **(B)**. **p < 0.01 vs. the sham group.

To further corroborate the effect of the blast-induced mTBI on the frontal cortex and hippocampus shown in MRS and ^18^F-FDG PET/CT examination, we employed histopathological analysis on brain tissue sections. Evans blue (EB) penetration assay showed significantly higher EB contents in mTBI rats at 1–3 h and 1 day after injury than those in the sham group (*p* = 0.0003, *p* < 0.0001, respectively), with no significant difference of EB contents between mTBI rats at 7 day and the sham group (*p* = 0.5252) (Figures 4A,B). This indicated that blast led to the disruption of the BBB immediately following injury to 1 day post-injury and recovered at 7 days post-injury. H&E staining showed no significant alteration in tissue composition of the frontal cortex (Figure 5A) and hippocampus (Supplementary Figure 3A) after blast at each time point. Furthermore, immunohistochemical staining for Iba 1, the microglia marker, revealed more Iba 1-positive cells in the frontal cortex of mTBI rats at each time point and elevated as the time went on (*p* < 0.0001), which was consistent with the findings of ^18^F-FDG PET and Ins alterations in MRS (Figures 5B,C). Glial changes in the frontal cortex were also among the most marked after blast-induced mTBI. GFAP immunostaining for astrocytes demonstrated the same trend as Iba 1 in the mTBI group (*p* < 0.0001) (Figures 5B,D). However, no marked difference was found on NeuN expression, the neuron marker, in the frontal cortex between the mTBI group and the sham group at each time point post-injury (*p* = 0.1025) (Figures 5B,E). On the contrary, no significant difference was shown between the mTBI group and the sham group on the expressions of Iba 1 (*p* = 0.8591), GFAP (*p* = 0.5935), and NeuN (*p* = 0.7671) in the hippocampus (Supplementary Figures 3B–E).

## Discussion

To the best of our knowledge, this is the first *in vivo* study to apply a combination of ^18^F-FDG PET and MRS to detect progressive changes of brain metabolism after blast-induced mTBI. We demonstrate early metabolic cerebral alterations following blast-induced mTBI, including hyperacute, acute, and subacute phases. Our current study reveals five main findings: (1) no visible brain injuries were observed on conventional T1- and T2-weighted imaging until 7 days following exposure to blast; (2) Ins, Glx, and Lac levels markedly elevated over time in the somatosensory cortex, while the Tau level immediately increased at 1–3 h and 1 day, and then returned to sham level on 7 days post-injury; (3) mTBI produced an acute increase in ^18^F-FDG uptake in the amygdala, somatosensory cortex, and motor cortex, but decreases in the midbrain structures; (4) Evans blue penetration assay revealed that blast led to the disruption of the BBB immediately following injury to 1 day post-injury and recovered at 7 days post-injury; and (5) histological findings showed a significant elevated expression of inflammatory marker Iba 1 and gliosis marker GFAP over time in the frontal cortex.

Traditionally, physicians have relied on CT and MRI to identify intracranial bleeding, lesions, and skull fractures. However, many patients with mTBI suffer neural injury at a microscopic level. They have persistent neurological symptoms despite normal MRI and CT imaging (Delouche et al., 2016; Honce et al., 2016). This study showed no visible sign of lesions in the brain on T1- and T2-weighted images following exposure to blast, which is similar to the previous findings (Tang et al., 2017). Because of the sensitivity of ^1^H MRS and ^18^F-FDG PET to metabolic changes *in vivo*, we performed these techniques to track the dynamic changes of brain metabolism following blast-induced mTBI.

Ins is a glial marker, including the reactive gliosis or microglia infiltration, which has been recognized as a marker of inflammation (Ashwal et al., 2004). We observed increased Ins level in the somatosensory cortex from 1–3 h to 7 days post-injury, consistent with the inflammation and gliosis responding to injury. These findings were confirmed by the immunohistochemistry using Iba 1 and GFAP immunolabeling in this study. Oxidative stress produces rapid and excessive elevation of extracellular glutamate and then is taken up by astrocytes and forms glutamine *via* the glutamine synthase enzyme, which is tightly controlled by gliosis (Zhong et al., 2006; Maas et al., 2008; Petronilho et al., 2010). Glx, combined glutamate–glutamine, also markedly increased over time after blast in this study. Several studies have detected elevated Glu levels that persist for several hours following severe TBI in humans and following mild to severe TBI in animal models (Singh et al., 2017), which agrees with our findings. Some other studies demonstrate that mitochondrial dysfunction caused by the very early stage of TBI may affect glutaminase activity leading to alterations of glutamate and glutamine (Xu et al., 2011). Lactate is the end product of anaerobic glycolysis and, therefore, a useful indicator of hypoxia/ischemic cellular condition. Lac level went up till 7 days post-injury, indicating hypoxia/ischemia and alternate metabolic pathways being likely activated in the somatosensory cortex after injury, which has also been reported in earlier studies on TBI (Makoroff et al., 2005; Dienel, 2012; Harris J.L. et al., 2012). Furthermore, Tau, a major osmolyte, immediately increased after trauma until 1 day post-injury, which may be due to the disruption of the BBB. In support of this idea, we performed Evans blue penetration assay to detect alteration of the BBB after injury, which revealed the disruption of the BBB immediately following injury to 1 day post-injury and recovered at 7 days post-injury. Moreover, recent studies have demonstrated that blast injury leads to the breakdown of the BBB immediately following injury with further increase in permeability continuing for several hours (Walls et al., 2016; Kuriakose et al., 2018). Interestingly, an elevated Cho level was only found on 7 days post-injury, which may be due to shearing of myelin and cellular membranes (Ross et al., 1998). The study of children with TBI showed significant increased Cho/Cr and decreased NAA/Cr in normal-appearing brain (Holshouser et al., 2005). However, no significant alterations were found in NAA level in our study. Immunohistochemistry showed that NeuN expression level did not change significantly over time, which confirmed the results of NAA in MRS.

Glucose is the major substrate for metabolism in the brain and has been studied extensively in non-blast TBI. Studies on non-blast TBI models reveal that metabolic alterations within the brain are dynamic and varied by regions depending on the severity of TBI, the model used, and the time of metabolic measurements (Richards et al., 2001; Casey et al., 2008; Harris N.G. et al., 2012; Prins et al., 2013). Few studies have detected changes in ^18^F-FDG uptake following primary blast exposure in rodents. Increased ^18^F-FDG uptake in the whole brain, especially regions associated with executive and vestibulomotor function, on 1 day post-injury was reported, and then resolved by 9 days following blast to head (Awwad et al., 2015). Jaiswal et al. (2019) examined ^18^F-FDG uptake in the brain by VOI- and voxel-based analysis following a single blast. Like us, they observed an acute increase in ^18^F-FDG uptake in the amygdala and somatosensory cortex and a decrease in multiple midbrain structures at 1–3 h following blast. Interestingly, in our current study, glucose metabolism in the motor cortex was also markedly elevated from 1 to 7 days post-injury, where increased Ins and Glx levels in MRS were observed. These temporal and regional changes in glucose metabolism following blast are due to inflammation and gliosis, which were confirmed by immunohistochemistry.

The hippocampus has been reported to be the common injured region. Previous studies have shown that the hippocampus was vulnerable to controlled cortical impact (CCI)-mediated neuronal death, occurring as early as 24 h post-injury and continuing for weeks after injury (Kabadi et al., 2012; Zhou et al., 2012; Loane et al., 2014). Rats undergoing lateral fluid percussion injury (FPI) showed markedly reduced FDG uptake in the hippocampus from 1 week after FPI to 6 months. However, in this study, no significant changes were found in the hippocampus on ^18^F-FDG PET, MRS, and immunohistochemistry from the hyperacute phase to the subacute phase. Consistent with our study, Jaiswal et al. (2019) showed no significant difference between the blast-induced mTBI group and the sham group in the hippocampus on ^18^F-FDG PET imaging at the acute and subacute stages. Walls et al. (2016) also showed no BBB damage and colocalized neuroinflammatory changes in the hippocampus from 4 h to several days post-injury in the blast-induced TBI model. These inconsistent results may be explained by different mTBI models and detected changes at different TBI stages. Further studies are needed to investigate alterations in the chronic phase and conduct other imaging modalities following blast-induced TBI.

In our study, ^18^F-FDG PET imaging reflects neuronal function and activity of cells including neurons and glial cells/inflammatory cells. MRS provides complementary information and assesses metabolites following injury, which can reflect the pathophysiological condition of the brain after injury indirectly, such as inflammation, ischemia, hypoxia, etc. ^18^F-FDG PET and MRS could be used to investigate neuropathological changes after brain injury from different aspects. Therefore, combining these two imaging methods could reflect more comprehensive neuropathological alterations *in vivo* after blast-induced mTBI. In addition, ^18^F-FDG PET provides metabolic information of the whole brain after a single scanning, while MRS offers metabolic information of one specific region. Thus, this may provide us a diagnostic strategy that ^18^F-FDG PET could firstly be performed on mTBI patients to find the abnormal regions and to define regions of interest for MRS, which may improve the diagnostic measures and therapeutic management after mTBI.

However, our study has limitations. First, the study design does not allow direct comparisons, since MRS and ^18^F-FDG PET were performed in two different cohorts. Second, we did not assess behavioral outcome of these animals. Future work should combine comprehensive behavioral testing with *in vivo* findings. Third, a small number of animals were used in the histological analysis. Fourth, the corpus callosum was not contained in the 58 regions of the W. Schiffer rat brain template, resulting in no information of ^18^F-FDG uptake in the corpus callosum. Previous studies have reported that the corpus callosum is more vulnerable to injury in non-blast TBI models (Brabazon et al., 2017). However, a characteristic pattern of blast-induced mTBI has not been identified. Thus, whether there are significant metabolic changes in the corpus callosum or not remains unknown. In future studies, other software should be used for PET image analysis, and DTI, sensitive to examining axonal injury, should also be performed to detect microstructural alterations after blast exposure.

## Conclusion

In conclusion, we demonstrate for the first time that a combination of ^18^F-FDG PET and MRS could detect progressive changes of brain metabolism after blast-induced mTBI in hyperacute, acute, and subacute post-injury period. Combining these two imaging methods could reflect more comprehensive neuropathological alterations *in vivo* after blast-induced mTBI. These findings offer useful information in understanding the pathophysiology of blast-induced mTBI, including inflammation, gliosis, hypoxia/ischemia, and BBB disruption.

## Data Availability Statement

The original contributions presented in the study are included in the article/Supplementary Material, further inquiries can be directed to the corresponding authors.

## Ethics Statement

The animal study was reviewed and approved by the Animal Use Subcommittee of Army Medical University.

## Author Contributions

XC, RJ, and KX conceived and designed the study. YL and CL established the model. YL, YG, and JZ collected and analyzed the MRI and MRS data. YT produced the PET radioligand. JS and FJ conducted the PET measurements. XC and JF analyzed the FDG PET data. HT and KL conducted and analyzed the histology. XC and KL performed the statistics, figure preparation, and wrote the manuscript. QZ edited the manuscript. All authors revised the manuscript and read and approved the submitted version.

## Conflict of Interest

The reviewer CZ declared a past co-authorship with one of the author FJ to the handling editor. The remaining authors declare that the research was conducted in the absence of any commercial or financial relationships that could be construed as a potential conflict of interest.
