# Supplementary material for: 18F-FDG PET Combined With MR Spectroscopy Elucidates the Progressive Metabolic Cerebral Alterations After Blast-Induced Mild Traumatic Brain Injury in Rats
Source: Front Neurosci. 2021 Mar 18;15:593723. doi: 10.3389/fnins.2021.593723 (PMC8012735; doi:10.3389/fnins.2021.593723)
Supplement: Supplementary file 1 [file Data_Sheet_1.docx]

Supplementary Material

| No. | Regions | Volume (mm^3^) | No. | Regions | Volume (mm^3^) |
| --- | --- | --- | --- | --- | --- |
| 1 | Accumbens_l | 7.944 | 30 | VisualCortex_r | 36.136 |
| 2 | Accumbens_r | 7.944 | 31 | HippocampusAnteroDorsal_l | 25.064 |
| 3 | Amygdala_l | 21.12 | 32 | HippocampusAnteroDorsal_r | 25.064 |
| 4 | Amygdala_r | 21.12 | 33 | HippocampusPosterior_l | 9.784 |
| 5 | Striatum_l | 43.552 | 34 | HippocampusPosterior_r | 9.784 |
| 6 | Striatum_r | 43.552 | 35 | Hypothalamus_l | 18.352 |
| 7 | AuditoryCortex_l | 27.52 | 36 | Hypothalamus_r | 18.352 |
| 8 | AuditoryCortex_r | 27.52 | 37 | Olfactory_l | 14.008 |
| 9 | CingulateCortex_l | 14.48 | 38 | Olfactory_r | 14.008 |
| 10 | CingulateCortex_r | 14.48 | 39 | ColliculusSuperior_l | 7.136 |
| 11 | EntorhinalCortex_l | 59.016 | 40 | ColliculusSuperior_r | 7.136 |
| 12 | EntorhinalCortex_r | 59.016 | 41 | Midbrain_l | 11.448 |
| 13 | FrontalAssociationCortex_l | 1.416 | 42 | Midbrain_r | 11.448 |
| 14 | FrontalAssociationCortex_r | 1.416 | 43 | VentralTegmentalArea_l | 5.528 |
| 15 | InsularCortex_l | 21.128 | 44 | VentralTegmentalArea_r | 5.528 |
| 16 | InsularCortex_r | 21.128 | 45 | Cerebellum_GM_l | 74.976 |
| 17 | MedialPrefrontalCortex_l | 6.304 | 46 | Cerebellum_GM_r | 75.008 |
| 18 | MedialPrefrontalCortex_r | 6.304 | 47 | Cerebellum_WM_l | 23.44 |
| 19 | MotorCortex_l | 32.608 | 48 | Cerebellum_WM_r | 23.56 |
| 20 | MotorCortex_r | 32.608 | 49 | ColliculusInferior_l | 5.744 |
| 21 | OrbitofrontalCortexl_l | 18.936 | 50 | ColliculusInferior_r | 5.744 |
| 22 | OrbitofrontalCortexl_r | 18.936 | 51 | Thalamus_l | 30.712 |
| 23 | ParACortex_l | 7.632 | 52 | Thalamus_r | 30.712 |
| 24 | ParACortex_r | 7.632 | 53 | Pituitary | 5.864 |
| 25 | RetrosplenialCortex_l | 18.92 | 54 | Cerebellum-blood | 5.064 |
| 26 | RetrosplenialCortex_r | 18.92 | 55 | CentralCanal-PAG | 9.904 |
| 27 | SomatosensoryCortex_l | 71.6 | 56 | Pons | 45.136 |
| 28 | SomatosensoryCortex_r | 71.6 | 57 | Septum | 9.36 |
| 29 | VisualCortex_l | 36.136 | 58 | Medulla | 59.544 |

Table S1. Regions in the W. Schiffer rat brain template and atlas

Notes: l, left hemisphere; r, right hemisphere.

Table S2. Comparisons of regional standardized uptake value ratios between the Sham and mTBI group.

Notes: SUVw of each region was presented as mean (standard deviation). Two-way analysis of variance (ANOVA) with repeated measures were used to analyze alterations of SUVw among mTBI and Sham groups at different time points. Two-tailed p values were calculated with the significance level at 0.05.

| Regions | Side | Sham | | | |  | mTBI | | | | P value |
| --- | --- | --- | --- | --- | --- | --- | --- | --- | --- | --- | --- |
|  |  | Baseline | 1-3h | 1 d | 7d |  | Baseline | 1-3h | 1 d | 7d |  |
| Amygdala | Left | 0.78 (0.02) | 0.76 (0.02) | 0.78 (0.01) | 0.76 (0.02) |  | 0.76 (0.02) | 0.80 (0.01) | 0.78 (0.02) | 0.76 (0.02) | 0.0033 |
|  | Right | 0.77 (0.04) | 0.76 (0.03) | 0.77 (0.03) | 0.77 (0.03) |  | 0.76 (0.02) | 0.81 (0.03) | 0.77 (0.02) | 0.75 (0.03) | 0.0445 |
| Somatosensory Cortex | Left | 1.06 (0.02) | 1.06 (0.02) | 1.05 (0.02) | 1.07 (0.03) |  | 1.05 (0.06) | 1.11 (0.04) | 1.11 (0.03) | 1.09 (0.03) | 0.0427 |
|  | Right | 1.05 (0.03) | 1.06 (0.03) | 1.04 (0.06) | 1.06 (0.05) |  | 1.06 (0.09) | 1.13 (0.07) | 1.10 (0.05) | 1.10 (0.05) | 0.0104 |
| Motor Cortex | Left | 1.08 (0.02) | 1.08 (0.02) | 1.08 (0.02) | 1.09 (0.02) |  | 1.07 (0.02) | 1.10 (0.02) | 1.12 (0.02) | 1.12 (0.02) | 0.0086 |
|  | Right | 1.07 (0.03) | 1.06 (0.04) | 1.08 (0.04) | 1.08 (0.04) |  | 1.08 (0.03) | 1.10 (0.03) | 1.12 (0.04) | 1.12 (0.03) | 0.0077 |
| Colliculus Inferior | Left | 1.08 (0.04) | 1.08 (0.02) | 1.08 (0.02) | 1.08 (0.02) |  | 1.07 (0.03) | 0.90 (0.06) | 0.94 (0.03) | 0.99 (0.02) | <0.0001 |
|  | Right | 1.08 (0.03) | 1.08 (0.04) | 1.09 (0.03) | 1.09 (0.05) |  | 1.09 (0.05) | 0.89 (0.04) | 0.96 (0.05) | 1.01 (0.05) | <0.0001 |
| Colliculus Superior | Left | 1.08 (0.04) | 0.90 (0.03) | 0.94 (0.03) | 1.00 (0.03) |  | 1.08 (0.04) | 1.08 (0.03) | 1.08 (0.02) | 1.08 (0.03) | <0.0001 |
|  | Right | 1.08 (0.04) | 0.89 (0.04) | 0.95 (0.04) | 1.01 (0.05) |  | 1.09 (0.03) | 1.07 (0.03) | 1.08 (0.04) | 1.08 (0.04) | <0.0001 |

**Supplementary Figures Legends**

Figure S1. Conventional structural magnetic resonance imaging (MRI) in mTBI rats. Representative T1- (up) and T2-weighted imaging (bottom) of MRI show no obvious abnormal intensity in brain regions from 1-3 h to 7 days after exposure to blast in mTBI rats.

Figure S2. Magnetic resonance spectroscopy (MRS) detects *in vivo* dynamic neurochemical alterations in the hippocampus region after blast-induced mTBI. Placement of voxel in the hippocampus region (white box, A) with a representative spectrum (up: mTBI 1-3 h post injury; bottom: sham) (B). (C-H) Box-plots show the relative levels of Ins, Glx, Lac, Tau, Cho, and NAA at 1-3 h, 1 day, 7 days post-injury in hippocampus of mTBI and sham rats. Box and whisker plots display median, first and third quartile, minimum, and maximum. The level of significance for intergroup differences was set at p < 0.05. No significant difference of Ins, Glx, Lac, Tau, Cho, and NAA were found between mTBI group and sham group. myo-inositol (Ins); taurine (Tau); choline (Cho); glutamine+ glutamate (Glx); glutamate (Glu); N-acetyl aspartic acid (NAA); lactate (Lac); creatine (Cr).

Figure S3. Immunohistochemical analysis of inflammation, injury, and astrogliosis in hippocampus after blast-induced mTBI. (A) H&E staining shows no obvious lesion after exposure to blast at each timepoint in mTBI rats. (B) Representative immunohistochemical staining for Iba 1, GFAP, and NeuN expression in hippocampus are shown at each timepoint following injury in sham and mTBI rats. (C-E) Iba 1, GFAP, NeuN expression levels were quantified. The level of significance for intergroup differences was set at p < 0.05. No significant difference of Iba 1, GFAP, and NeuN expression were found between mTBI group and sham group. The scale bar denotes 100 μm in (A), 50 μm in (B).
