# Supplementary figures and images for: 18F-FDG PET Combined With MR Spectroscopy Elucidates the Progressive Metabolic Cerebral Alterations After Blast-Induced Mild Traumatic Brain Injury in Rats
Source: Front Neurosci. 2021 Mar 18;15:593723. doi: 10.3389/fnins.2021.593723 (PMC8012735; doi:10.3389/fnins.2021.593723)

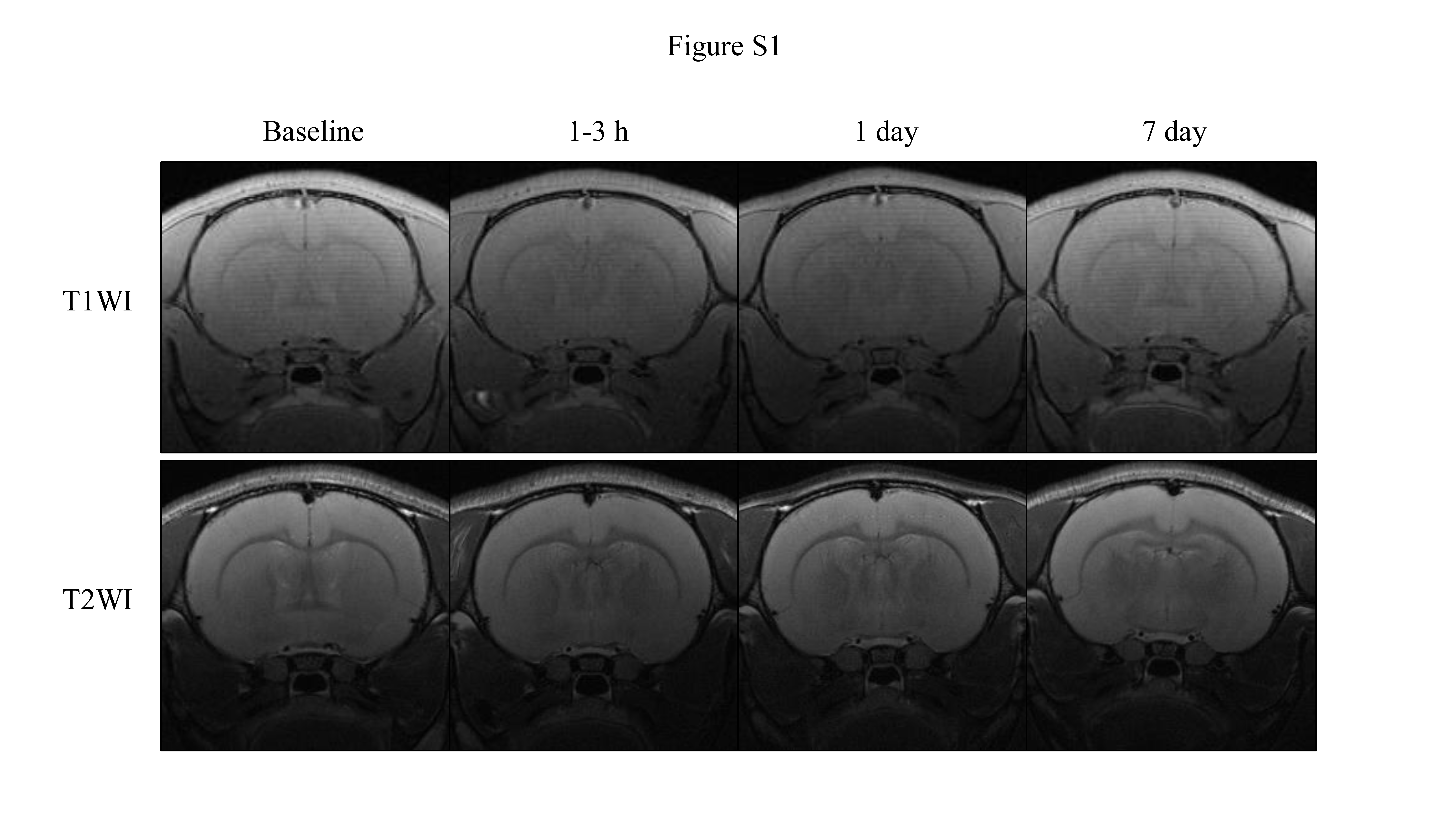

Supplement: Supplementary file 2 [file Image_1.TIFF]

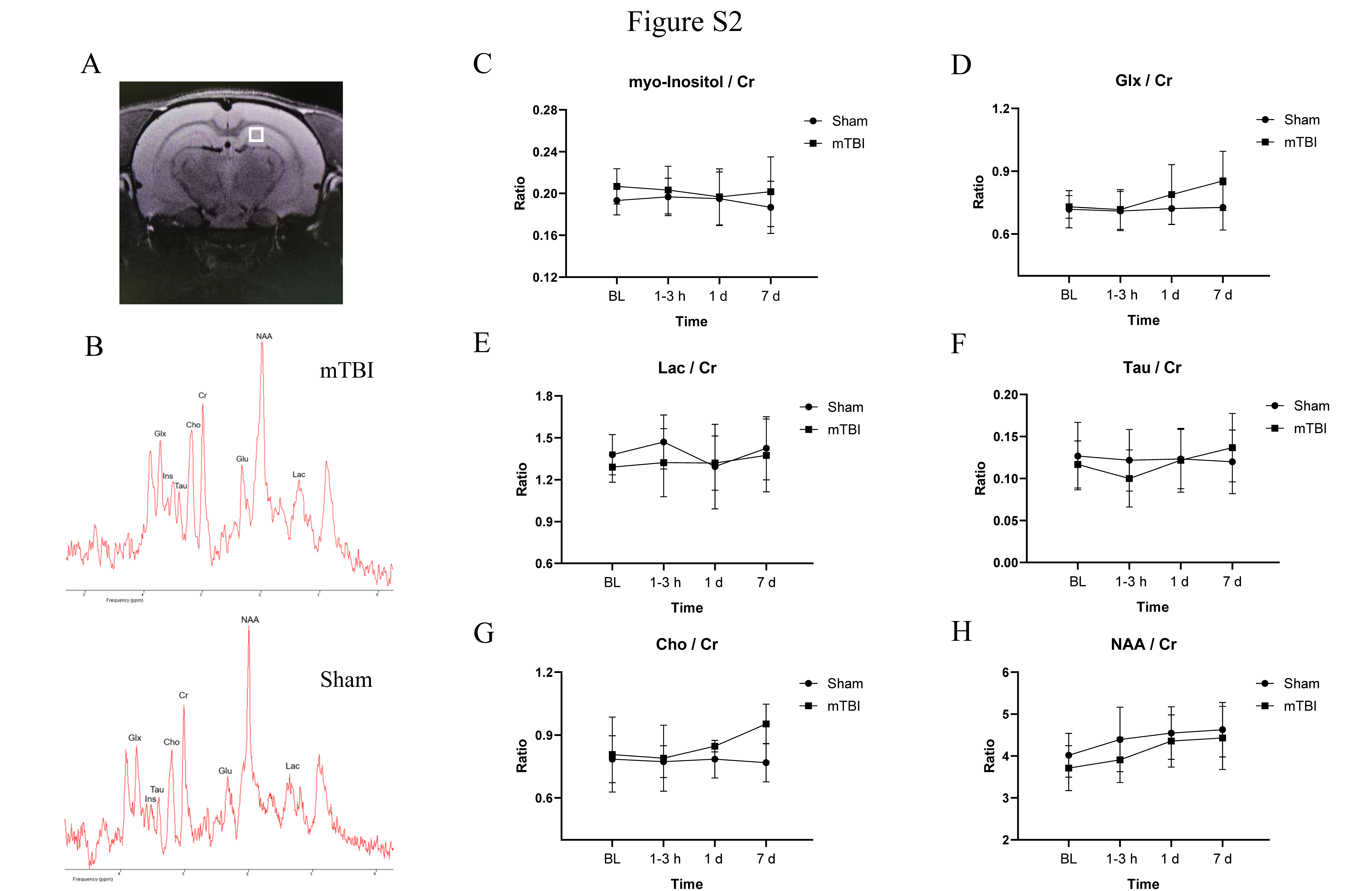

Supplement: Supplementary file 3 [file Image_2.TIFF]

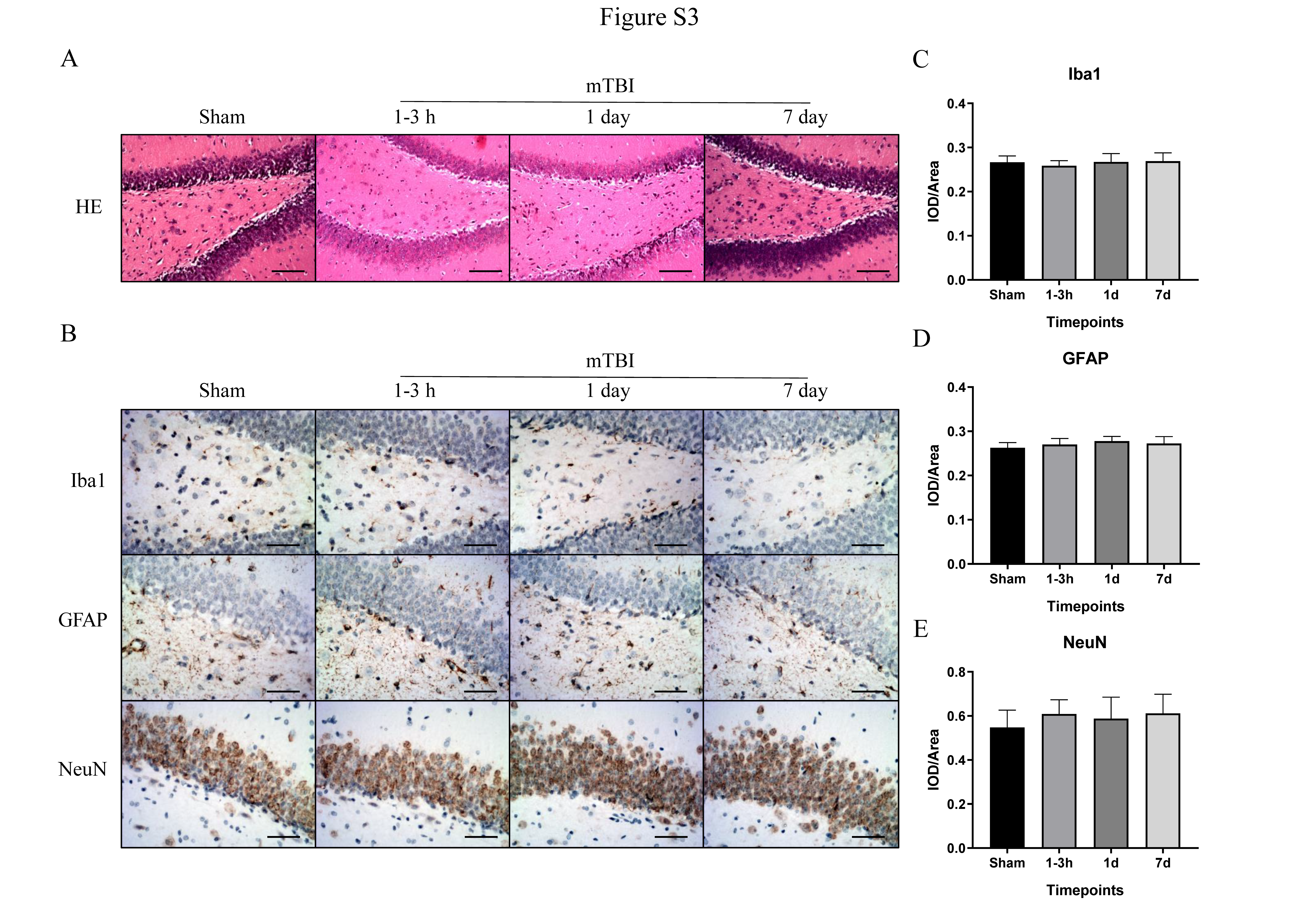

Supplement: Supplementary file 4 [file Image_3.TIFF]
